# Curcumin Effects on Memory Impairment and Restoration of Irregular Neuronal Distribution in the Hippocampal CA1 Region After Global Cerebral Ischemia in Male Rats

**DOI:** 10.32598/bcn.9.10.365

**Published:** 2019-09-01

**Authors:** Leila Kamali Dolatabadi, Masoumeh Emamghoreishi, Mohammad Reza Namavar, Hamze Badeli Sarkala

**Affiliations:** 1. Department of Neuroscience, School of Advanced Medical Sciences and Technologies, Shiraz University of Medical Sciences, Shiraz, Iran.; 2. Department of Pharmacology, School of Medicine, Shiraz University of Medical Sciences, Shiraz, Iran.; 3. Research Center for Psychiatry and Behavior Science, Shiraz University of Medical Sciences, Shiraz, Iran.; 4. Clinical Neurology Research Center, Shiraz University of Medical Sciences, Shiraz, Iran.; 5. Histomorphometry and Stereology Research Center, Shiraz University of Medical Sciences, Shiraz, Iran.; 6. Department of Anatomy, School of Medicine, Shiraz University of Medical Sciences, Shiraz, Iran.

**Keywords:** Curcumin, Global cerebral ischemia, Memory, Neurons

## Abstract

**Introduction:**

Global Cerebral Ischemia (GCI) causes neuronal damage with subsequent neurological and cognitive impairments. Curcumin has anti-inflammatory, antioxidant, and neuroprotective properties, which makes it a potential candidate for improving GCI-induced impairments. This study aimed to investigate the effects of curcumin on the neurological and memory deficits, as well as spatial neuronal distribution in the Cornu Ammonis 1 region after GCI in rats.

**Methods:**

56 Sprague-Dawley male rats were randomly assigned into 4 groups of sham (n=14), control (n=14), curcumin 50 mg/kg (n=14), and curcumin 100 mg/kg (n=14). Each group was divided into the two subgroups of short-term (7 days) and long-term (28 days) treatment periods. The Neurological Severity Score (NSS), passive avoidance task, and the traction test were performed at postoperative days of 0, 1, 2, 3, 7, 14, 21, and 28. The novel object recognition test and Voronoi tessellation were carried out on days 7 and 28 after GCI.

**Results:**

Curcumin 100 mg/kg significantly decreased neurological severity score on postoperative days of 7 and 28 compared with the control (P<0.001) and curcumin 50 mg/kg groups (P<0.05–P<0.001), **respectively**. Also, curcumin 100 mg/kg significantly increased step-through latency times on postoperative days of 3–28 and 14–28 compared with the control (P<0.05–P<0.001) and curcumin 50 mg/kg groups (P<0.01–P<0.001). Moreover, it increased the novelty preference index during the novel object recognition test in the 28-day treatment subgroup after GCI. Curcumin (100 mg/kg) could maintain the neuronal aggregation in the CA1 region after GCI at a level near to what is generally observed in normal rats.

**Conclusion:**

Curcumin could improve memory and neurological deficits and restore irregular neuronal distribution in the CA1 region after GCI in a time-dependent manner, and its higher dose was more effective than its lower dose. Curcumin may have beneficial effects on reducing brain complications after ischemia.

## Highlights

- Curcumin can be a potential candidate for improving memory and neurological problems after global cerebral ischemia.
- A high dose of curcumin¬ (100¬ mg/kg) and its long-term administration (28 days) significantly decreased neurological severity score after global cerebral ischemia.
- A high dose of curcumin¬ (100¬ mg/kg) and its long-term administration (28 days) significantly increased step-through latency time under passive avoidance task and the novelty preference index during the novel object recognition after global cerebral ischemia.
- A high dose of curcumin¬ (100¬ mg/kg) and its long-term administration (28 days) could maintain the neuronal aggregation in the CA1 hippocampal region after global cerebral ischemia.

## Plain Language Summary

Transient Global Cerebral Ischemia (GCI) is a cerebrovascular condition dramatically reducing cerebral blood flow leading to a selective and delayed pyramidal neuronal death in the hippocampal CA1 area. Herbal medications and their active ingredients are a potential source for finding new remedies to improve cognitive and neurological deficits that occur following GCI. Several studies supported the anti-inflammatory, antioxidant, and neuroprotective effects of curcumin as an active constituent of turmeric. Because of its biological properties, curcumin is an appropriate candidate for improving injuries after brain ischemia. This study is an attempt to assess the acute and chronic effects of curcumin on the spatial neuronal distribution in CA1 area and on the neurological symptoms and memory impairment after GCI. Experiments were conducted on 56 adult male Sprague Dawley rats. Based on the results, a high dose of curcumin (100 mg/kg) and its long-term administration (28 days) showed a greater effect on memory function, neurological deficits, and regular neuronal aggregation in the CA1 hippocampal region after GCI.

## 1. Introduction

Brain ischemia is a major cause of disability and the second leading cause of mortality worldwide (Li, Shalabi, Ji, & Meng, 2017). Transient Global Cerebral Ischemia (GCI) is a cerebrovascular condition dramatically reducing cerebral blood flow leading to a selective and delayed pyramidal neuronal death in the hippocampal Cornu Ammonis 1 area (Kiryk et al., 2011). Hippocampal damage is associated with cognitive impairments observed in rodents and humans following GCI (Gulinello, Lebesgue, Jover-Mengual, Zukin, & Etgen, 2006).

Therapeutic regimens to recover from ischemic injuries are still scarce (Attari et al., 2016; Girbovan, Kent, Merali, & Plamondon, 2016). Recent studies have focused on discovering new agents that can enhance hippocampal neurogenesis. These agents should possess antioxidant, antiapoptotic, or neuroprotective properties to overcome or limit the consequences of neural damage, and consequently prevent long-term cognitive impairments after brain ischemia (Attari et al., 2016; Gulinello et al., 2006).

Herbal medications and their active ingredients are a potential source for finding new remedies to improve cognitive and neurological deficits that occur following brain ischemia. Several studies supported the anti-inflammatory, antioxidant, and neuroprotective effects of curcumin as an active constituent of turmeric (Shah et al., 2016). Because of its biological properties, curcumin is an appropriate candidate for improving injuries after brain ischemia. Previous studies support the beneficial effects of curcumin on neurogenesis, neuronal differentiation, neuroprotection, and improvement of cerebral pathological changes following brain ischemia (Attari et al., 2016; Liu et al., 2016; Shah et al., 2016; Zhao et al., 2010). However, only a few studies have reported the effects of curcumin on neurological and cognitive impairments after focal brain ischemia (Tu et al., 2014; Zhao et al., 2010) without mentioning GCI.

Besides, the earlier studies have mainly focused on evaluating the acute effects of curcumin, i.e. immediately or up to 7 days after the induction of brain ischemia. Thus, there is no study on the long-term effects of treatment with curcumin and its time course effects on neurological and memory impairments following focal or GCI. Also, neuronal death in the CA1 area of the hippocampus has been already attributed to cognitive impairments after GCI (Wang, Xue, Zhao, Gao, & Wei, 2012), but there is still no study on evaluating the neuronal aggregation following brain ischemia and the effect of curcumin treatment. Voronoi tessellation is a systematic way to characterize cell aggregation and provides information about the spatial distribution of cells in a specific area (Duyckaerts & Godefroy, 2000; Jiao, Berman, Kiehl, & Torquato, 2011).

The objectives of this study were three-fold. First to examine the time-course effects of long-term treatment with curcumin on the neurological symptoms and memory impairment, to evaluate the spatial distribution of the neurons in CA1 area following GCI, and finally to assess the acute and chronic effects of curcumin on the spatial neuronal distribution in CA1 area all after GCI.

## 2. Methods

### 2.1. Animals

A total of 56 adult male Sprague Dawley rats (weight: 200–250 g) were prepared from Shiraz University of Medical Sciences. The rats were kept under a 12:12 h light: dark cycle at 22±2°C with ad libitum feeding. Rats were acclimatized to their new conditions for one week before the study. All study procedures were done following the National Institute of Health guideline (1985).

### 2.2. Study design

The Sprague-Dawley rats were randomly assigned into four groups of sham (n=14), control (n=14), curcumin 50 mg/kg (n=14), and curcumin 100 mg/kg (n=14). Each group was subdivided into two subgroups of short-term (7 days) and long-term (28 days) treatment periods. Two treatment groups were given curcumin (Merck, Germany) orally dissolved in Phosphate Buffered Saline (PBS). The control group received 4 mL/kg PBS orally, and the sham group received nothing. A single daily dose was administered (16–24 hours after operation) for 7 days in the short-term subgroup and 28 days in the long-term treatment subgroup. The curcumin dosage was determined based on a pilot study and previously published works (Liu et al., 2014).

### 2.3. Induction of global cerebral ischemia

Transient GCI was induced by using the two-vessel occlusion model (Kakkar, Muppu, Chopra, & Kaur, 2013). After 12 hours of fasting, the rats were anesthetized using 100 mg/kg ketamine and 10 mg/kg xylazine; and two common carotid arteries were ligated using small artery clips for 20 min after separating the vagus nerve. The ischemia duration was chosen based on a pilot study and previous works. After 20 min, the clips were removed from both common carotid arteries, and restoration of blood flow was visually confirmed. The rats whose pupils were not dilated and responsive to light and showed seizures were excluded from the experiments (Kakkar et al., 2013). In sham-operated rats, anesthesia and operations were performed, but common carotid arteries were not clamped.

### 2.4. Assessment of neurological symptoms

The Neurological Severity Score (NSS) was evaluated according to a previously described stroke index as follows: No neurological impairment: 0; Hunched posture: 1; Ptosis: 2; Circling behavior: 3; Splayed-out hind limb: 4; and Epileptic seizures: 5. The highest score indicates the most serious damage (Dai et al., 2013). The NSSs were assessed 6 hours after completing perfusion (postoperative day 0) and then, on postoperative days 1, 3, and 7 for short-term subgroup and days 1, 3, 7, 14, 21, and 28 for long-term treatment subgroup (Dong et al., 2016). On the first postoperative day (16–24 hours after operation), drug administration was begun, and evaluations were performed before, 1 and 3 hours after administration.

### 2.5. Traction test

Locomotor function was evaluated by the traction test, 15 minutes before step-through inhibitory avoidance task. Traction apparatus was a metallic wire (2 mm diameter, 60 cm length) stretched horizontally between two wooden vertical planes 50 cm above the floor. The rats were first trained to remain on the wire for at least 180 s. The time a rat took to remain suspended on the wire was recorded on postoperative days 1, 3, and 7 for short-term treatment subgroup and on days 1, 3, 7, 14, 21, and 28 for long-term treatment subgroup (Dong et al., 2016; Ahmadi-Mahmoodabadi, Nasehi, Emam-Ghoreishi, & Zarrindast, 2016).

### 2.6. Step-through inhibitory avoidance task

The 2-way shuttle box (BorjSanaat Co., Tehran, Iran) consisted of two light (white opaque plexiglas, 20×20×30 cm^3^) and dark (black opaque plexiglas, 20×20×30 cm^3^) compartments connected to each other by a sliding door (8×8 cm^2^). Foot shock (Intensity: 0.7 mA; Frequency: 50 Hz; Duration: 3 s) was delivered through rods of stainless steel grids on the floor of the dark compartment.

In the habituation trial, the rats were gently placed in the light compartment. 15 seconds later, the sliding door was opened to let the animals enter the dark compartment. When the rat completely entered the dark compartment, the sliding door was closed, and the rat was immediately returned to its home cage. Animals that waited for more than 120 s to enter into the dark compartment were excluded from the experiment. Thirty minutes later, the acquisition trial was performed. In this stage, when the rat entered into the dark compartment, the sliding door was closed, and a foot shock was instantly delivered to the grid floor of the dark compartment. After 15 s, the rat was temporarily returned to its cage and tested again two minutes later. In case of entering the dark compartment, foot shock is given for the second time. The maximum training trial for each rat was three times. The retention or retrieval test was performed 24 hours later in a manner like the acquisition trial, but the electrical shock was not delivered to the grid floor. The time to enter the dark room (Step-through Latency (STL)) was recorded as an inhibitory avoidance memory. A cutoff time of 300 s for STL measurement was set for all animals that remained in the light room. The STL time was recorded on postoperative days 1, 3, and 7 for short-term treatment subgroup and on days 1, 3, 7, 14, 21, and 28 for long-term treatment subgroup (Ahmadi-Mahmoodabadi, Nasehi, Ghoreishi, & Zarrindast, 2016).

### 2.7. Novel object recognition test

Recognition memory was assessed by the Novel Object Recognition Test (NORT) (Noorafshan, Karimi, Kamali, Karbalay-Doust, & Nami, 2017). On days 5 and 26, after the operation, the rats in the 7-day and 28-day treatment subgroups were habituated respectively for 20 min in an empty white box with no roof (100×100×40 cm^3^). The following day, two similar colored cubes made of compact plastic were placed on the box floor, and rats were allowed to explore the objects for 3 min (the training phase). To evaluate the short-term memory, we replaced a new metallic gray cylinder (5 cm in diameter and 10 cm in height) with one of the colored cubes 19 minutes later. Then, the rats were allowed to explore it for 3 min. 24 hours later (day 7 or day 28 after operation) and 2 hours after the traction test, the long-term memory was evaluated by replacing one of the familiar objects with a novel object different from the previous one, letting rats explore it for 3 min. In all phases, the total time that rats spent with the novel and familiar objects were recorded. The preference for exploration of novel objects was used as an index for a successful recognition memory test. The Novelty Preference Index (NPI) was calculated as follows: (time spent exploring the novel object - time spent exploring the familiar object)/(time spent exploring the novel object + time spent exploring the familiar object).

### 2.8. Preparation of brain sections

After performing behavioral tests on days 7 and 28 after the operation, the rats’ brains in the short-term and long-term treatment groups were fixed by transcardial perfusion of 0.1 M PBS (pH 7.4) followed by 4% paraformaldehyde in PBS. Brains were removed and postfixed overnight and then transferred to 30% sucrose solution in 0.1 M PBS and kept at 4ºC for 48–72 hours (Dai et al., 2013). The samples were then kept at −80ºC. 40 μm thick coronal sections of the brains were cut by Leica CM1860 cryostat (Leica Microsystems, Solms, Germany) and stored in a cryoprotectant solution at −20ºC (Larsson, Lindvall, & Kokaia, 2001) until using for cresyl violet staining. The staining was performed according to the protocol provided by the manufacturer (Merck) with minor modifications. The slides were then placed in the following solutions consecutively, 95% alcohol for 1 min, 70% alcohol for 3 min, distilled water for 5 min, cresyl violet for 40 min, distilled water for 3 min, 70% alcohol for 3 min, 95% alcohol for 5 s, 100% alcohol for 5 s, butanol I for 2 min, butanol II for 2 min, xylol I for 2 min, and xylol II for 2 min. Afterward, the sections were cover-slipped with entlan glue.

### 2.9. Evaluation of spatial distribution of neurons

The spatial distribution of neurons in the CA1 region of the hippocampus was evaluated with the Voronoi tessellation method, which was obtained by constructing Voronoi polygons. Each polygon encompasses the areas in which the cells are accumulated and close together (Torquato, 2013). Thus, a polygon area indicates the spaces that a cell occupies. The area and the number of closest Voronoi polygons to each other were then obtained (Jiao et al., 2011). To draw the Voronoi polygon diagram, brain sections were analyzed using the video-microscopy system. The CA1 microscopic images were taken with a 40x to1800x objective lens. The favored parameters were the neuron’s nuclei. Each image was imported to the ImageJ software. After setting the scale of the images, we marked the neurons by clicking on their nuclei. In the next step, the polygons were designed by running “Plugins” then “Analyze” and finally “Voronoi” orders. Black and white images were taken from the tessellated areas with changing the threshold. Finally, the areas of polygons were measured by selecting “Analyze” and then “Measure” (Safaeian & David, 2013; van Horssen et al., 2014).

### 2.10. Statistical analysis

The obtained data are presented as the Mean±SEM. The Kolmogorov-Smirnov test was performed to determine the normality of data distribution. Differences in NSS, STL time, and latency to fall time were assessed by repeated measures ANOVA by considering the time as a within-subjects factor and the treatment as a between-subjects factor. The least significant difference test was used as a post hoc test for comparing the mean differences between groups. If there was an interaction between time and treatment, 1-way ANOVA followed by Tukey’s post hoc test were performed to evaluate the mean differences between groups at each time. The NORT data were analyzed by 1-way ANOVA, followed by Tukey’s post hoc test for comparing means between groups. The approximate power of each analysis was computed with alpha level of 0.05. The statistical analyses were performed in SPSS V. 21 (SPSS Inc., Chicago, Illinois, USA). The statistical significance level was set as P<0.05.

## 3. Results

### 3.1. Effect of curcumin on neurological severity disorder

**Figure 1.**
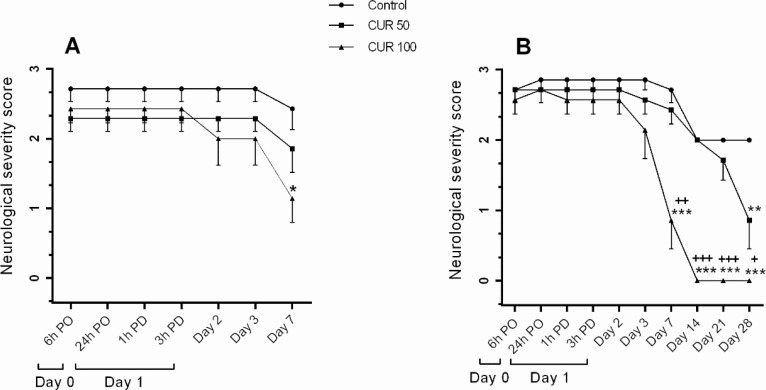
Data represent Mean±SEM of NSS in rats received vehicle (control) A. Curcumin 50 mg/kg (CUR50) or curcumin 100 mg/kg (CUR100) for 7; B. 28 days after GCI. The NSS was evaluated on different postoperative (PO) days i.e. day 0 (6 h PO), day 1 (24 h PO, 1 h after drug administration [PD] and 3 PD), days 2, 3, 7, 14, 21, and 28 PO ^*^P<0.05; ^**^P<0.01; ^***^P<0.001 significantly different from the control group +P<0.05; ++P<0.01; +++P<0.001 significantly different from CUR50

Since the sham group had no neurological deficit (score: 0) throughout the study period, it was excluded from the statistical analysis. In the 7-day treatment subgroup, repeated measures ANOVA indicated the significant effect of time (P<0.001), no significant interaction between time and treatment, and a trend toward the significant impact of treatment (P=0.088). However, pairwise comparisons of means showed a significant difference between curcumin 100 mg/kg and control groups (P=0.039). Post hoc tests revealed a significantly lower NSS in curcumin 100 mg/kg group compared with the control group at postoperative day 7 (P<0.05) (Figure 1A).

In the 28-day treatment subgroup, repeated measures ANOVA results demonstrated a significant effect of time (P<0.001), treatment (P<0.001), and time × treatment interaction (P<0.001). Post hoc tests indicated a significant reduction in NSS for the curcumin 100 mg/kg group compared with the control and curcumin 50 mg/kg groups on postoperative days 7, 14, 21, and 28 (P<0.05–P< 0.001) (Figure 1B). There was also a significantly lower NSS in 50 mg/kg curcumin-treated group on the postoperative day 28 compared with the control group (P<0.01) (Figure 1B).

### 3.2. Effect of curcumin on passive avoidance task and locomotor function

**Figure 2.**
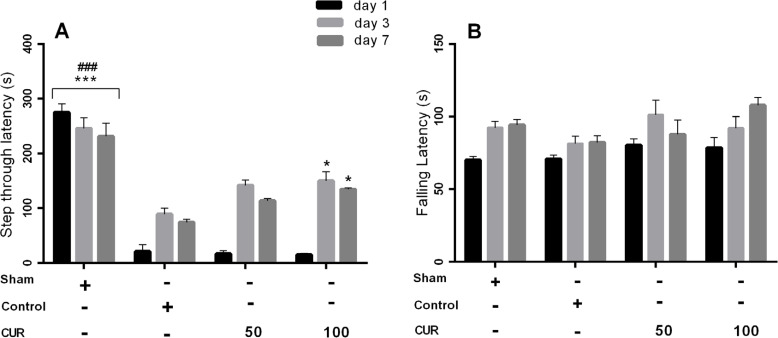
Bars represent Mean±SEM A. STL time; B. Falling latency time (s) at days 1, 3, and 7 after GIC in the sham group and in rats received vehicle (control), curcumin 50 mg/kg (CUR50), or curcumin 100 mg/kg (CUR100) for 7 days ^*^P<0.05; ^***^P<0.001 significantly different from the control group ###P<0.001 significantly different from control, CUR50, CUR100

Repeated measures ANOVA showed the significant effect of time (P<0.001), treatment (P<0.001), and time × treatment interaction (P<0.001) on STL time in the 7-day treatment subgroup. The groups of control, curcumin 50 mg/kg, and curcumin 100 mg/kg, had significantly shorter STL times compared with the sham group at all study days (P<0.001). However, the STL time was significantly higher in curcumin 100 mg/kg group compared with the controls on postoperative days 3 and 7 (P<0.05) (Figure 2A). Repeated measures ANOVA showed a significant effect of time (P<0.001), but the treatment and time × treatment interaction had a significant impact on latency to fall times (Figure 2B). The latency to fall time significantly increased on postoperative days 3 and 7 following operation compared with the time recorded on the first postoperative day (P<0.001).

**Figure 3.**
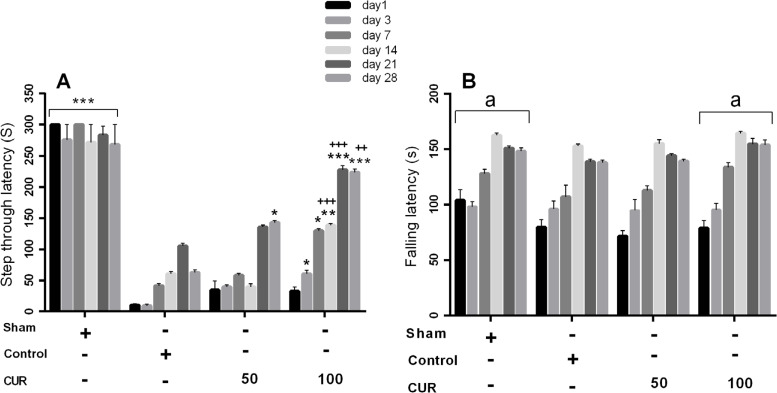
Bars represent Mean±SEM A. STL time; B. Falling latency time (s) at days 1, 3, 7, 14, 21, and 28 following GCI in sham group and in rats received vehicle (control), curcumin 50 mg/kg (CUR50), or curcumin 100 mg/kg (CUR100) for 28 days ^*^P<0.05; ^**^P<0.01; ^***^P<0.001 significantly different from the control group +P<0.05; ++P<0.01; +++P<0.001 significantly different from CUR50 ^a^ P<0.01: The mean of total latency to fall time throughout the study period significantly different from the control group

In the 28-day treatment subgroup, repeated measures ANOVA results showed a significant effect of time (P<0.001), treatment (P<0.001), and time × treatment interaction (P<0.01) on STL time. Post hoc comparisons at each studied time revealed that the curcumin 100 mg/kg group had significantly longer STL times on days 3 (P<0.05), 7 (P<0.05), 14 (P<0.01), 21 (P<0.001) and 28 (P<0.001) compared with the control group and on days 14 (P<0.001), 21 (P<0.001), and 28 (P=0.01) compared with the curcumin 50 mg/kg group (Figure 3A). Besides, the STL time was significantly longer in the group treated with curcumin 50 mg/kg compared with the control group on days 21 (P<0.05) and 28 (P=0.01)(Figure 3A). The sham group showed significantly longer STL time compared with the samples in the groups of curcumin 50 mg/kg, curcumin 100 mg/kg, and control on days 1, 3, 7, 14, and 21 after the operation (P<0.001). On day 28, after the operation, the sham group showed a significantly longer STL time compared with the curcumin 50 mg/kg and control groups (P<0.001).

In the 28-day treatment subgroup, the results showed the significant effects of time (P<0.001) and treatment (P<0.01), but the time × treatment interaction had no significant effect on the latency to fall time. Significantly longer times for latency to fall were observed in the sham and curcumin 100 mg/kg groups compared with the controls (P<0.01) and curcumin 50 mg/kg group (P<0.01). Furthermore, the latency to fall times significantly increased on days 3 to 28 compared with the first day (P<0.001) and decreased on days 21 and 28 compared with day 14 (P<0.001).

### 3.3. Effect of curcumin on the novel object recognition test

**Figure 4.**
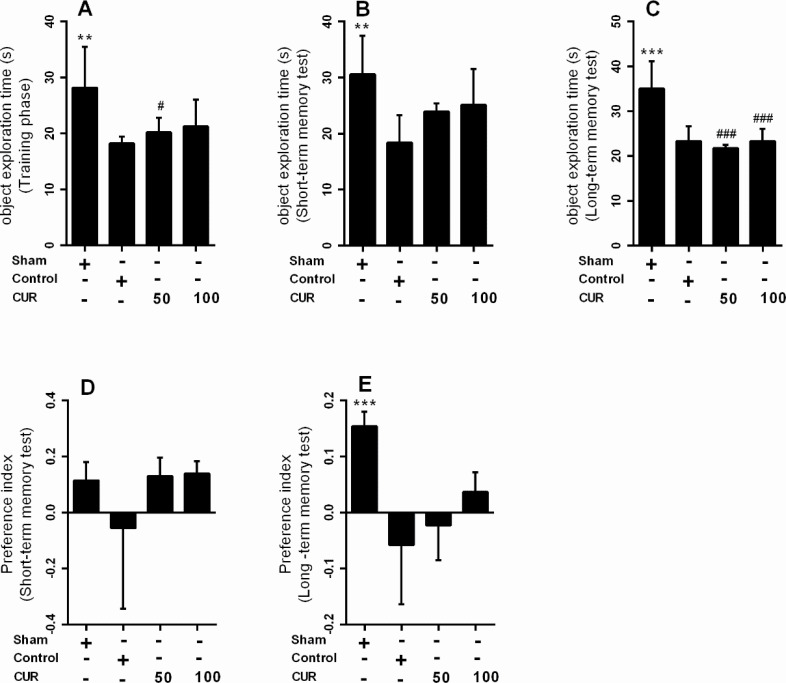
Box plots represent Mean±SEM of exploration times (n=7/group) A. The novel objects in training; B. Short-term; C. Long-term memory; D. Phases, novelty preference index for short-term; E. Long-term memory in NORT for the sham group and in rats received vehicle (control), curcumin 50 mg/kg (CUR50), or curcumin 100mg/kg (CUR100) for 7 days after GCI ^*^P<0.05; ^**^P<0.01; ^***^P<0.001 significantly different from the control group #P<0.05; ##P<0.01; ###P<0.001 significantly different from the sham group

In the 7-day treatment subgroup, there were no significant differences between NPI of the study groups in long-term memory test (Figure 4D); however, there was a significant reduction in NPI value in the control (P<0.001) and curcumin 50 mg/kg (P<0.01) groups compared with the sham group in long-term memory test (Figure 4E).

**Figure 5.**
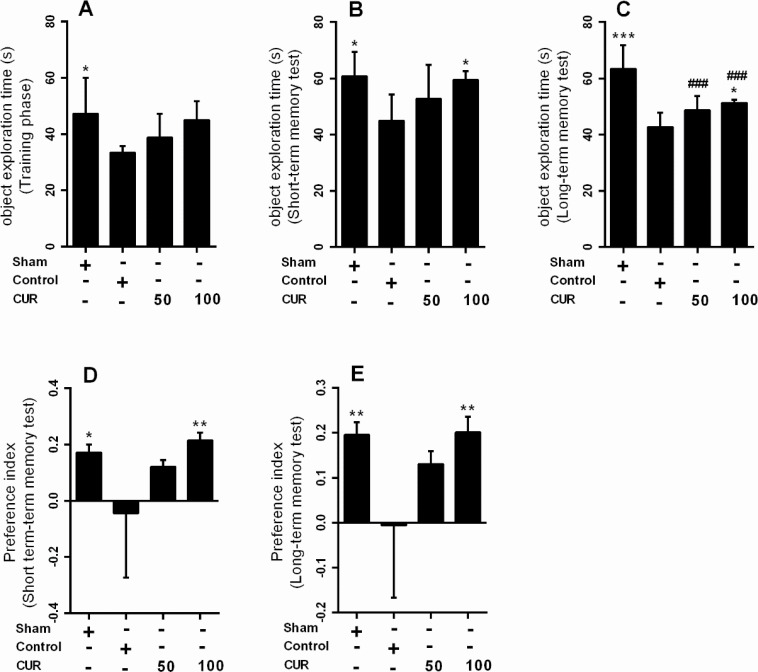
Box plots represent Mean±SEM of exploration times (n=7/group) A. The novel objects in training; B. Short-term; C. Long-term memory; D. Phases, novelty preference index for short-term; E. Long-term memory in NORT for the sham group and rats received vehicle (control), curcumin 50 mg/kg (CUR50), or curcumin 100mg/kg (CUR100) for 28 days after GCI ^*^P<0.05; ^**^P<0.01; ^***^P<0.001 significantly different from the control group #P<0.05; ##P<0.01; ###P<0.001 significantly different from the sham group

In the 28-day treatment subgroup, the NPI in short-term (P<0.05) and long-term memory (P<0.01) tests were significantly lower in the control group than in the sham group (Figure 5D and 5E). Moreover, the curcumin 100 mg/kg significantly increased the NPI in short-and long-term memory tests compared with the control group (P<0.01) (Figure 5D and 5E). Linear regression analysis showed that the effect of curcumin on NPI in short-term (Mean±SEM=0.129±0.038; P=0.003) and long-term memory (Mean±SEM=0.103±0.03; P=0.003) was dose-dependent; i.e. an increase in the curcumin dosage increases its effect on the recognition memory.

### 3.4. Effect of curcumin on the spatial distribution of neurons in the CA1 area

**Figure 6.**
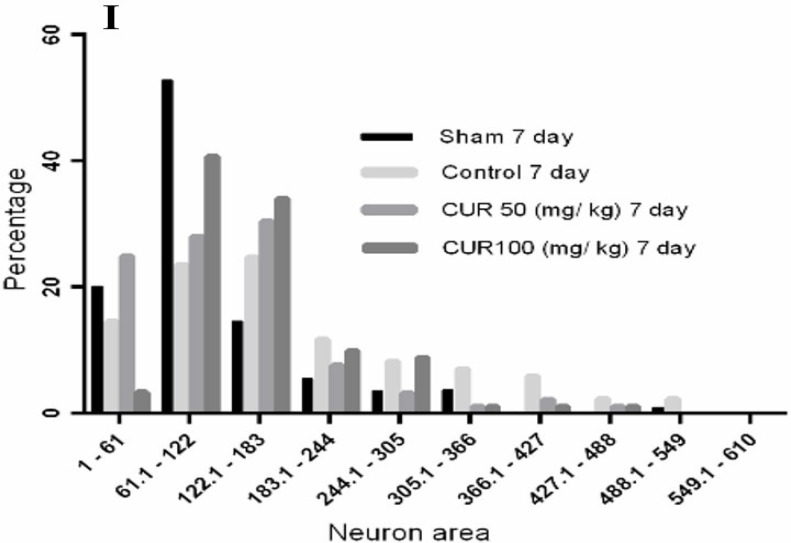
A micrograph of neurons in the CA1 for the groups A. Sham; C. Control; E. Curcumin 50 mg/kg (CUR50); G. Curcumin 100 mg/kg (CUR100) in the 7-day treatment subgroups obtained from Voronoi tessellation method. After setting the scale, neuronal nuclei were marked, and the polygons were superimposed on them using the ImageJ software (B, D, F, H, respectively). Lower bar graph (I) indicates the percentage of polygon distribution classified into various categories according to the polygon areas (μm2)

Figure 6 shows the Voronoi polygon areas calculated for the CA1 neuron’s nuclei in the 7-day treatment subgroup. In the sham group, approximately 52.7% of the polygon areas were within the range of 61–122 μm^2^, while 23.5%, 28%, and 40.7% of the polygon areas ranged between 61 and 122 μm^2^ in the control, curcumin 50 mg/kg, and curcumin 100 mg/kg groups, respectively. Moreover, 20.1% and 27.2% of polygons in the sham group were stretched out within the range of 1–61 μm^2^ and 122–610 μm^2^, respectively. The areas of polygons within the range of 1–61μm^2^ were 14.6%, 25%, and 3.4%; and within the range of 122–610 μm^2^ were 61.9%, 47% and 55.9% for control, curcumin 50 mg/kg and curcumin 100 mg/kg groups, respectively.

**Figure 7.**
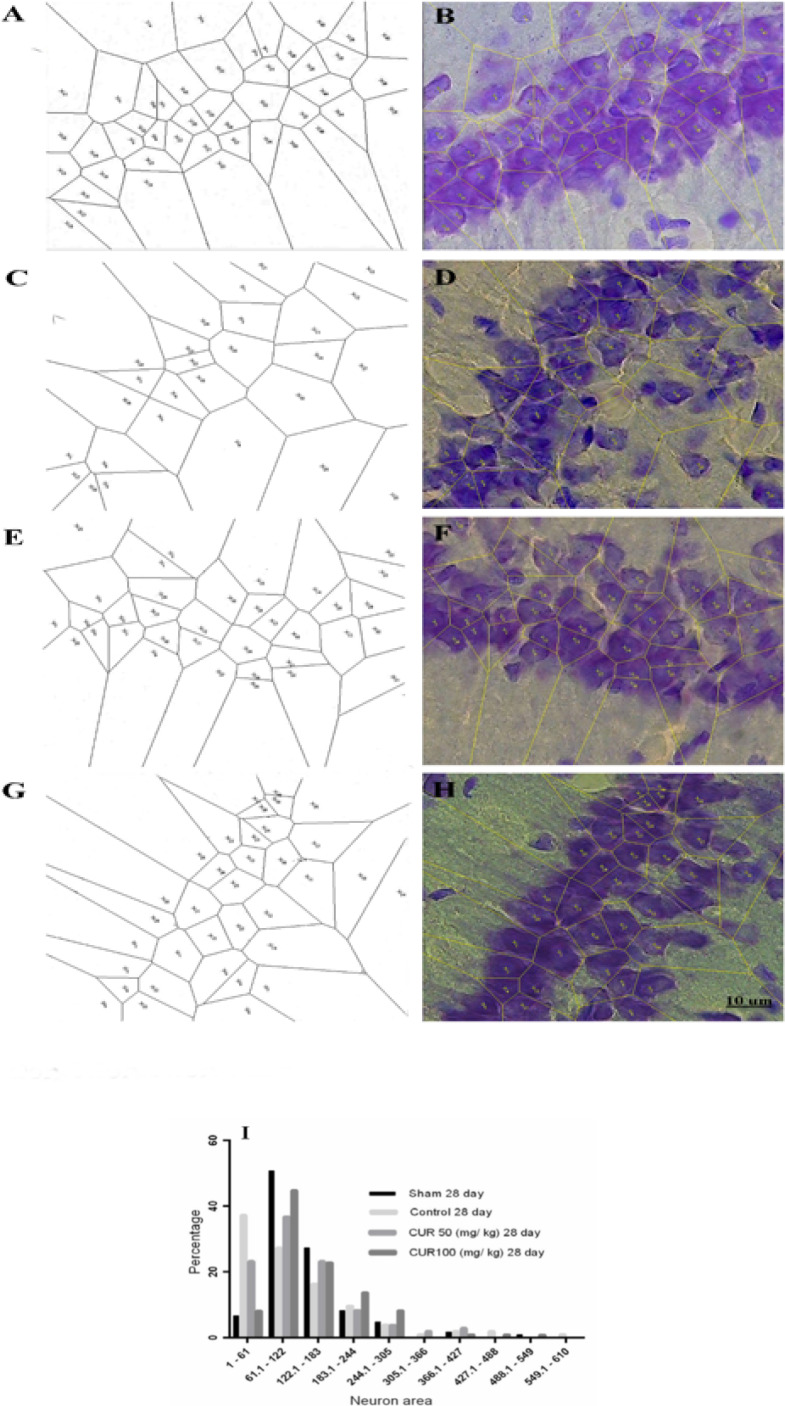
A micrograph of neurons in the CA1 for the groups A. Sham; C. Control; E. Curcumin 50 mg/kg (CUR50); G. Curcumin 100 mg/kg (CUR100) in the 7-day treatment subgroups obtained from Voronoi tessellation method. After setting the scale, neuronal nuclei were marked, and the polygons were superimposed on them using the ImageJ software (B, D, F, H, respectively). Lower bar graph (I) indicates the percentage of polygon distribution classified into various categories according to the polygon areas (μm^2^)

In the 28-day treatment subgroup, 50.6%, 27.3%, 36.7%, and 44.7% of the polygons areas were found within the range of 61–122 μm^2^ in the sham, control, curcumin 50 mg/kg and curcumin 100 mg/kg groups, respectively. Moreover, the percentages of polygon areas for the sham, control, curcumin 50 mg/kg and curcumin 100 mg/kg groups were 6.6%, 37.2%, 23.3%, and 8.1%, within the range of 1–61 μm^2^ and 42.8%, 35.5%, 40%, and 47.2% within the range of 122–610 μm^2^, respectively (Figure 7 A–I).

## 4. Discussion

The present study revealed that curcumin could improve neurological deficit and memory impairment and protected the normal distribution of neurons in the hippocampal CA1 region after GCI in a time-dependent manner. The beneficial effects of curcumin mainly started on day 7 after GCI and reached its highest level on day 28 post GCI. With a higher dosage (100 mg/kg), curcumin was more effective than with the lower dosage.

The results indicate that curcumin improves neurological deficits following GCI. A high dose of curcumin is more effective in improving neurological symptoms; i.e. it could completely recover neurological injury and accelerate the recovery period. To the best of our knowledge, there is no other time-course study on the effect of curcumin on neurological symptoms after GCI to be compared with our results. However, there is a report that curcumin with 100 mg/kg dose (not 50 mg/kg dose) could significantly reduce NSS 24 hours after focal ischemia (Yang, Zhang, Fan, & Liu, 2009). In our study, on the contrary, 100 mg/kg curcumin could not significantly decrease NSS 24 hours after GCI. This discrepancy may be due to the difference in the used models of ischemia (global vs. focal ischemia).

Curcumin could improve memory after GCI, with a higher dose exerting better efficacy on memory restoration than its lower dose. Moreover, curcumin, even at a higher dose, could not completely revive impaired memory after GCI, because its maximum effect was not comparable to that of the sham group. To the best our knowledge, there is no other study on the impact of curcumin on memory impairment after GCI; however, previous reports have indicated that curcumin can improve memory in sleep-deprived rats (Noorafshan, Karimi, Karbalay-Doust, & Kamali, 2017), Alzheimer mice (Pan, Qiu, Lu, & Dong, 2008), rats with colchicine-induced cognitive impairment (Kumar, Naidu, Seghal, & Padi, 2007), and rates with 3-nitropropionic acid-induced cognitive impairment (Kumar, Padi, Naidu, & Kumar, 2007). These reports agree with our study results that curcumin can ameliorate memory impairment following GCI.

The improved avoidance memory observed after using curcumin does not seem to be because of the effect of curcumin on locomotor function. Improved motor function over time in all study groups might indicate that the rats learned how to maintain their balance on the wire. Moreover, their overall motor function following GCI had improved by curcumin with a near-normal level. The reported time-course effects of curcumin on motor function agree with the improved NSSs, suggesting that the curcumin effect on motor function mirrored the recovery from the neurological deficit, but it had no direct effect on motor function. This finding is supported by the previous studies reported that curcumin could not affect locomotor activity (Zhao et al., 2012; Zhu et al., 2014). In any case, the improved memory observed after treatment with curcumin cannot be related to the effect of curcumin on locomotor function.

In this study, the improving effect of curcumin on memory was further supported by the findings that long-term treatment with curcumin can fully restore recognition memory following GCI. We found no study that has evaluated the effect of curcumin on the impaired recognition memory following GCI; however, consistent with our findings, some studies have reported that 21-day administration of curcumin can improve memory acquisition in aged male mice (Woolley, Marsden, Sleight, & Fone, 2003) and sleep-deprived rats subjected to the novel object-recognition task (Noorafshan et al., 2017).

In our study, the tessellation diagrams illustrate that GCI can cause an irregular neuronal distribution and a large gap between neurons in the CA1 region within the range of 61–122 μm^2^. The decreased neuronal aggregation may be due to either neuronal death or inflammation and indicates the inability of auto-recovery processes in reinstating the normal distribution of neurons. Curcumin could preserve normal aggregation of neurons in the CA1 region, depending on its dose and treatment duration. The antioxidant, anti-inflammatory, or neuroprotective properties of curcumin have likely led to neuronal survival after GCI. We suggest that these mechanisms be addressed in future studies.

The exact mechanism of curcumin in improving memory and reducing neurological deficit following GCI is still unknown; however, the antioxidant, anti-inflammatory, neuroprotective, and neurogenesis properties of curcumin may contribute to its beneficial effects after GCI. Time-dependent effects of curcumin on memory and NSS suggest that curcumin can exert its effects possibly through adaptive mechanisms that need time, including neurogenesis, altering synaptic plasticity or gene expression. The curcumin likely improves memory after GCI by increasing neurogenesis. This notion is in agreement with a recent study that showed that long-term administration of curcumin promoted neurogenesis and improved cognition in aged rats (Dong et al., 2012). The neuroprotective effect of curcumin has been reported in focal ischemia (Li, Tan, Yu & Gang , 2013; Wang et al., 2012; Zhuang, Lin, Song, & Li, 2009) and GCI (Huang et al., 2011; Attari et al., 2016). These reports, consistent with our findings that curcumin could maintain the normal neuronal distribution in the CA1 region after GCI, suggest that neuroprotective and neurogenesis properties of curcumin can be related to its improving effects on the neurological deficits and memory impairments following GCI. We recommend that these mechanisms be addressed in future studies.

Curcumin improves memory function and neurological deficits, and maintain regular neuronal aggregation following GCI in a time-dependent manner. A high dose of curcumin (100 mg/kg) and its long-term administration (28 days) showed a greater effect on reviving GCI-induced impairments. Curcumin can be a potential candidate for improving memory and neurological problems after GCI.

## Ethical Considerations

### Compliance with ethical guidelines

The local Ethics Committee of Shiraz University of Medical Sciences and approved the study (Ethical code: IR.SUMS.REC.1395.S541).
